# Path and Bayesian network analyses in the complex design of a well-being survey via New Zealand’s Integrated Data Infrastructure

**DOI:** 10.3389/fpsyt.2026.1826907

**Published:** 2026-07-28

**Authors:** Irene Suilan Zeng, Junxiang Hao, Mandie Foster, Kelly Marie Jones

**Affiliations:** 1 Biostatistics, School of Clinical Sciences, Faculty of Health and Environmental Sciences, Auckland University of Technology, Auckland, New Zealand; 2 National Institute of Stroke and Neuroscience, Faculty of Health and Environmental Science, Auckland University of Technology, Auckland, New Zealand

**Keywords:** Bayesian network, complex survey analysis, integrated data infrastructure, path analysis, quantile regression, replicate weights, triangulation in analytical models

## Abstract

**Introduction:**

In mental health research involved with sensitive features and privacy issues, using integrated data offers an efficient alternative to traditional approaches such as interviews or in−person data collection. These conventional methods frequently face logistical barriers, including low response rates among study populations. Using integrated data from multiple existing administrative and survey sources provides protection for participants and economic savings for researchers, despite being constrained by the limitations of the original data sources. Our research questions were: 1) Can we conduct path and network analyses of school absenteeism, psychosocial factors, and mental health outcomes using the integrated survey data from New Zealand’s Integrated Data Infrastructure (IDI)? and 2) How can we account for the complex design of the General Social Survey (GSS) to ensure representative inference?

**Materials and methods:**

The study population was New Zealand youth enrolled in school, aged 15 years and older in 2018, who participated in the 2018 GSS. The study analyzed the Ministry of Education data integrated with the 2018 GSS survey data from New Zealand’s IDI. To explore the relationship between outcomes of including the WHO-5, “perceived life worthwhileness”, “perceived general health”, and predictors including school absenteeism, psychosocial factors, and family factors, quantile mixed−effects regression, path analysis, and Bayesian network (BN) analysis were used. The complex survey design was calibrated using replicated weights from the GSS, a design-based method for complex sampling, in path and regression analyses, and bootstrap resampling, in BN analysis.

**Results:**

In descending order from the weighted path model, overall life satisfaction (standardized path coefficient: 0.36), perceived health condition (0.34), ease in accepting cultural identity (0.12), and trust in the education system (0.07) were all significantly (positively) related to students' mental health well-being (WHO-5). Perceived life worthwhileness correlated with the WHO-5 but was only connected to overall life satisfaction. Similar factors were identified for perceived health, with additional attributing factors, such as fear of crime in the area and the family’s overall well-being.

**Conclusion:**

The integration of Bayesian networks and path analysis offers rigorous methods for detecting complex interrelationships among integrated mental health outcomes from population data and variables from a complex survey design.

## 1 Introduction

Integrated data have gained popularity in health research due to their benefits relating to feasibility, efficiency, and completeness of high-dimensional information. In mental health research, where privacy is a paramount concern, integrated datasets provide a practical alternative to resource−intensive methods such as face−to−face interviews or primary data collection in observational studies, which often suffer from low response rates. Using aggregated, rather than individual, data from multiple existing databases provides a cost-effective approach to research for the public good while minimizing participant burden and ensuring confidentiality. However, integrated data are limited in terms of what data have already been collected, their quality and content, and whether the data will be available for future research. Rigorous analytic methods are required to maintain the validity of the integrated data when analyzing high−dimensional information. Integrated data analysis can be guided by recent hybrid/complementary learning frameworks that combine supervised and unsupervised learning to study complex systems. Together with the traditional triangulation framework for internal validity based on the convergence from multiple methods, the integrated data analysis achieves better validity by applying complementary models.

In high-dimensional spaces, sparse regression, path, and network structure analyses are commonly used methods when there are many variables present with a small number of subjects. *Bayesian networks* (BNs) are considered efficient graphical models used in surveys and health-related studies. BNs are widely applicable in health outcome studies to identify risk factors associated with diseases related to cardiovascular disease, oncology, and other important public-health domains (1, 2). In the epidemiology of mental health, probabilistic dependencies between psychological symptoms, social factors, and behavioral variables can be mapped using BNs to enable the identification of critical paths influencing depression, anxiety, and other psychiatric outcomes (3). Complementing BNs, path analysis (as part of covariance structure modeling) allows researchers to specify and test *a priori* directional relationships among multiple correlated variables.

Within New Zealand’s integrated data infrastructure, under the Five Safes data governance principle (4), administrative data can be integrated with survey data based on probability linking. We integrated education and health administrative data with the 2018 General Social Survey (GSS) to examine the relationships between school absenteeism, psychosocial and family factors, and students’ mental health well−being.

Our research questions were 1) To what extent could we conduct path and network analyses of school absenteeism, psychosocial factors, and mental health outcomes using the integrated survey data from New Zealand’s Integrated Data Infrastructure (IDI)? And (2) How could we account for the complex design of the GSS to ensure representative inference?

## 2 Literature review

### 2.2 Methodological triangulation and internal validity

To strengthen internal validity, methodological triangulation from multiple analytical methods and data sources has been suggested by researchers across biostatistics, epidemiology, sociology, and other areas of science.

The earliest advocate of this approach, Denzin (5), introduced the methodological triangulation framework in sociological methods. Subsequently, triangulation across methods with differing susceptibility to confounding factors and measurement errors became a recognized strategy for strengthening causal inference in epidemiology and biostatistics (6, 7). Consistency of findings across complementary analyses increases confidence that the observed associations are not attributable to method-specific biases (77). In 2018, Munafò and Davey Smith (8) proposed a strategy of protecting flawed ideas by triangulating different approaches for answering one research question. The conclusions drawn from agreed-upon results from different approaches were found to enhance the reproducibility and internal validity.

Recent developments in biostatistics and data science include the complementary hybrid learning framework, which combines unsupervised learning and supervised learning methods (9). The idea aligns with Breiman’s (10) two culture about complementing data modeling and algorithmic models for a research question. The former are hypothesis-driven, and the latter are pragmatic, with an increasing use in big data.

### 2.3 Supervised learning and regression-based inference

Supervised learning could be hypothesis-driven with pre-specified outcomes, and directional and non-directional relationships among outcomes and variables of interest. Conventional supervised learning methods include statistical models of parametric regressions, such as linear/non-linear, and non-parametric regressions, such as quantile regression and tree-based methods (11). Path analysis (12–14) is used to test hypothesized relationships between a set of continuous observed variables modeled in terms of systems of equations. It will estimate pre-specified structural relationships between outcomes and explanatory variables, with specified relationships among explanatory variables simultaneously tested in the structural model.

Supervised learning uses labeled mental health data, such as clinician-rated depression scores, diagnostic categories, or symptom severity, to train models that can predict outcomes. This includes both classification (e.g., predicting the presence of a disorder) and regression (predicting continuous symptom scores) (15). Supervised methods such as random forests, logistic regression, gradient boosting, and decision trees have been used by researchers to predict outcomes, including suicidal behavior/risk, personality traits, symptom severity, and bipolar episodes, and/or to generate clinical risk models (15–18).

Xu et al. (19) used ordinary least squares (OLS) regression to display an overall mean model. They further examined the regression relationship between the variables (demographic characteristics, contact with family, and social support) and depressive disorders in 3,156 college students in China using quantile regression. They used conditional quartiles (P25, P50, and P75) from prior studies as proxies to describe the results of their quantile regression analysis (20). While OLS detected a significant association between regular contact with family and average depressive disorder, quantile regression identified a significant effect at the 25th quantile, where students with milder depression levels notably benefited from regular family contact. Here, quantile regression provided additional insight into providing more adaptable, meaningful interventions for students.

Sun et al. (21) used a path analysis approach to identify the mediation association of meaning in life (direct and indirect) with depression, hopelessness, and suicidal ideation among 90 adults experiencing major depressive disorders in Taiwan. The path analysis demonstrated that depression was the most significant factor in relation to suicidal ideation, and meaning in life showed an indirect mediating effect on suicidal ideation through a decrease in depression. A path analysis approach can clearly identify a direct/indirect relationship among variables (22).

### 2.4 Unsupervised learning and Bayesian network analysis

Unsupervised learning does not require pre-specified outcome variables. Examples are cluster analysis and network analysis (11).

Bayesian networks have gained popularity in recent decades for their ability to find compact representations of structural relationships among a large number of variables *(*23–25*)*. BNs, or probabilistic graphical models (directed acyclic graphs), are used to model dependencies between variables and their joint probability distribution (26). In surveys and health-related studies, BNs are increasingly being used to identify complex interdependencies and underlying causality between observed data (27). BNs apply both graphical modeling techniques and probabilistic methods according to Bayes’ theorem to establish interdependencies between predictors and target outcomes (28). One significant benefit of using BNs lies in their graphical representation of the interdependencies between survey and health-related variables in a graphical model, which makes understanding and interpretation easier (29). BNs have also increased in popularity in survey analysis to outline pivotal elements or relationships in complex datasets. For example, to determine which service features pointed to maximum satisfaction, Cugnata et al. (30) used BNs to evaluate customer satisfaction surveys. In BNs, the frequency trends of an arc (the linkage between two nodes in a network) are viewed across numerous graphical model structures to test indicator–outcome patterns. Sensitivities are assessed to test changes in model prediction based on input elements. These methods have shown both the strength and reliability of data illustrations in survey analysis (30).

In public health and epidemiological research, BNs have been applied to model interconnections among risk factors, demographics, and health outcomes, such as the incidence of chronic diseases (31). These models allow researchers to identify influential predictors and simulate intervention effects. For example, BNs can help to inform the design of targeted prevention strategies by quantifying how modifiable risk factors interact probabilistically (32). BNs have also been employed to analyze how early life experiences, socio-economic difficulties, and environmental factors evolve together to impact long-term mental health outcomes. For example, mental health issues during adolescence were predicted using a BN to identify probabilistic relationships between family instability, school engagement, peer relations, and early behavioral manifestations (33).

BNs are powerful tools for analyzing multi-source survey and administrative data (34). However, drawing valid inferences from such complex data requires consideration of survey design and weighting. Ignoring design features, such as stratification or clustering, can distort uncertainty estimates, undermining the credibility of network-based conclusions.

### 2.5 Use of replicated weights for network analysis in complex survey data

Complex survey datasets frequently represent stratified multistage designs involving unequal probabilities of selection and non-independent samples (35). To estimate parameters along with standard errors that adjust for design effects related to complex sampling schemes, weighting methods are specifically designed to provide unbiased survey variances of estimators (36). One common solution, a design-based method introduced to correct complex survey variance estimation, is to use “replicated weights”. Essentially, survey data users are provided “a set of alternative weights” or “replicates” that represent a set of alternative weights for a dataset or perturbations against which to test the original weights (37). In this way, the use of weights simplifies variance calculations, as survey data users only re-analyze the data using the weights to check “the variation across those replicates to create an estimate”, reflective of sampling error introduced by the complex survey design (38). Many large datasets (such as those published by statistical agencies or “OECD studies like PISA”) provide data files with replicated weight values, which statistical computer packages enable users to utilize. Typically, statistical agencies provide dozens of *replicated weight sets* within a single dataset, which conventional computer analysis procedures allow users to apply to estimate design-consistent standard errors (39).

The usefulness of incorporating replicated weights in network or multivariate analyses of survey data has been widely recognized in the literature (40). When researchers estimate relationships among variables using BNs, correlation networks, or regression-based path analysis, ignoring the complex survey design can lead to the underestimation of uncertainty and overconfidence in statistical significance (41). Replicated weights enable design-based inference accounting for the survey’s stratification, clustering, and weighting structure (42). The general principle involves performing a series of analyses using “pseudo-samples” constructed to reflect the original sampling design. Each replication provides an alternative version of the dataset, allowing variability across replicates to represent sampling uncertainty in variance estimation (43).

A well-documented example was found in studies using the Programme for International Student Assessment (PISA) data. Oberski (2014) applied structural equation modeling (SEM) to examine how self-efficacy and self-concept influence mathematics performance, incorporating latent and observed variables. The PISA dataset included 80 replicated weights designed to produce accurate standard errors. When these replicated weights were incorporated, the estimated standard errors were approximately 38% larger than those obtained without accounting for the survey design, demonstrating that naive analyses can significantly overstate precision and yield misleadingly narrow confidence intervals (44). Using replicated weights, which commonly provide larger variances, there were applications (45) that showed narrower variances in seasonally adjusted series data. The replicates could more accurately reflect the smoothing and model-based adjustment (46).

This reasoning directly applies to BN modeling in survey-based research. Marella and Vicard (47) used a bootstrapping Bayesian network to produce a pseudo-population based on the sampling design and a selection probability. The pseudo-population was generated using a plug-in approach (48, 49), which is another adaptation approach compared to *ad hoc* rescaling and bootstrap sampling. It is based on the idea of expanding the sample to a pseudo-population to predict the original one and drawing bootstrap samples from such a pseudo-population according to an appropriate resampling design. Replicated weights were also used, similarly to bootstraps, to assess the robustness and stability of model topology, allowing for a credible interpretation of dependencies between observed and latent variables. For example, Barbe and Bertail (50) used heuristic random reweighting for each observation in the sample.

Various software provides functions to include replicated weights when deriving variances and estimators (e.g., means, totals, ratios, and regression coefficients). This software includes R (using the survey and lavaan.survey packages), SAS (PROC SURVEYREG and PROC CALIS), and STATA (44). These methodologies enable the easier integration of design-based variance estimation methodologies into complex models.

The use of replicated weights becomes more useful in an integrated system, such as New Zealand’s IDI, which combines complex survey data with administrative data. Models related to pathways concerning education to employment outcomes or modeling various sociological health determinants need to incorporate replicated weights to generalize results appropriately (51). In the mental healthcare setting, replicated weights can also play a crucial role when investigating population-wide trends concerning service use patterns and unmet needs. For example, to link well-being indicators identified via surveys with administrative information on mental health service utilization, replicated weights can be used to make design-consistent estimates about disparities concerning help-seeking behavior, and to trace out pathways to persistent unmet mental health needs (52).

In conclusion, using replicated weights in either network or multivariate analyses ensures that model uncertainty parameters or standard errors are unbiased with respect to the survey design data. This increases both methodological rigor and inferential validity (53).

The existing literature presents few studies that consider using triangulation between different analytical models, such as using supervised and unsupervised statistical learning methods to draw convergence in results and identify differences from different models. Even fewer studies consider the complex design of population surveys in these analyses or adjust the results using survey weights.

The current study proposes using a supervised learning–quantile regression model for a skewed outcome variable (the mental health well-being index—WHO-5) and a repeatedly collected exposure variable (school absenteeism) with other explanatory variables of interest. For comparison, the current study also proposed a supervised structure learning–path analysis covariance structure model and an unsupervised learning–Bayesian structure learning model for the WHO-5 and the other well-being outcomes (“perceived life worthwhileness” rating and perceived subjective well-being) in response to the first research question. Furthermore, the complex survey structure was calibrated using replicated weights provided in New Zealand’s IDI data, integrating a National GSS with education system data to assess the mental health well-being of students for the second research question.

We organized the sections into the following: Section 3, the different methods used; Section 4, results from the integrated GSS; Section 5, simulations; and Section 6, conclusion.

## 3 Materials and methods

### 3.1 Study population

**Figure 1:**
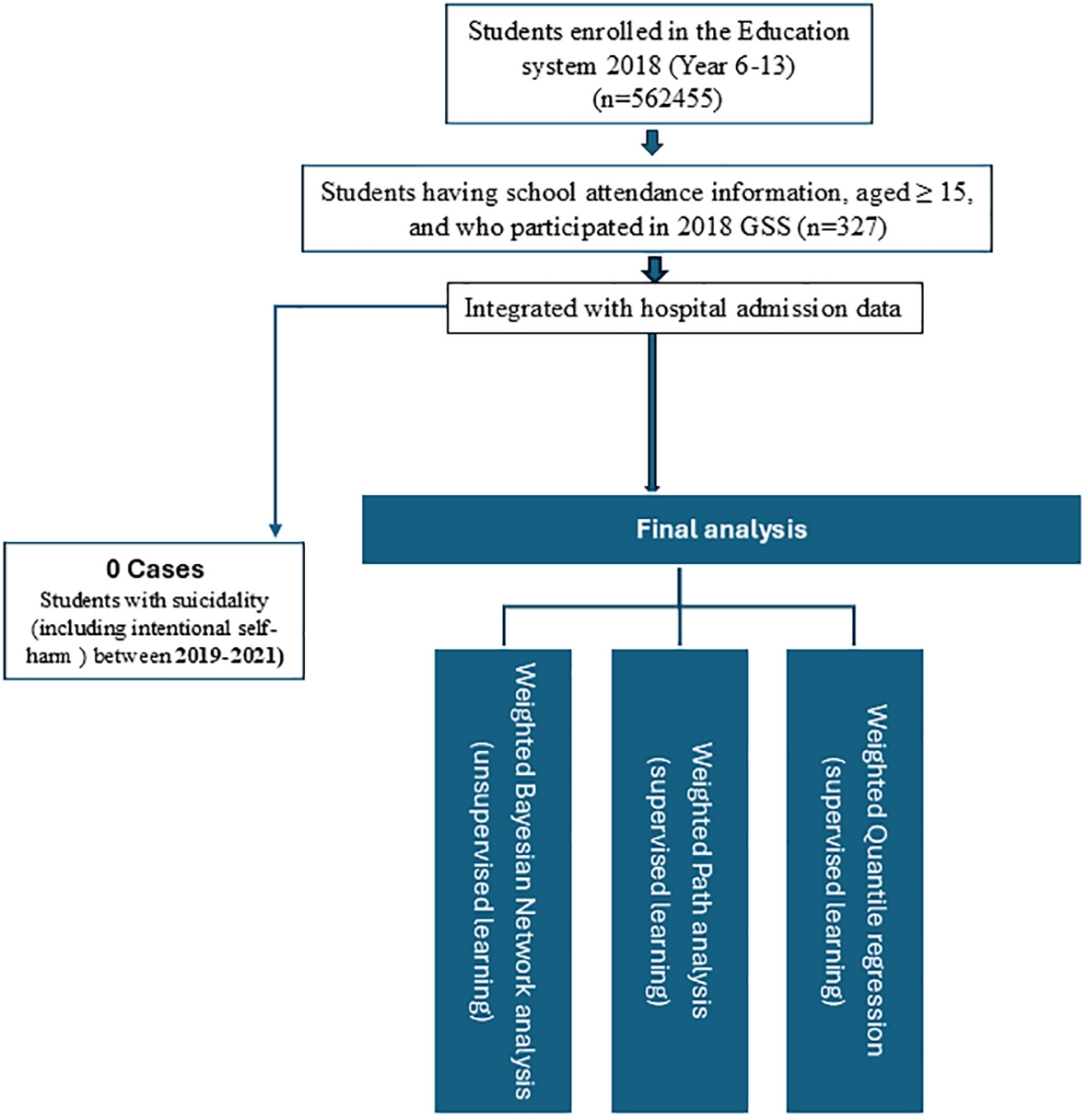
Study design flow chart.

The study population consisted of New Zealand youth enrolled in year levels 6–13 in 2018 who participated in the 2018 GSS (**Figure 1**). The GSS’s target population is usually the resident population aged 15 years and older in private dwellings on the North Island, South Island, or Waiheke Island of New Zealand.

### 3.2 Integrating school data and GSS 2018 survey data

The study integrated the Ministry of Education data with the 2018 GSS data within New Zealand’s IDI, a national research facility. The IDI holds de-identified microdata about people and households. The data comprise life events, health, education, income, social benefits, migration, and justice, which are integrated to form the IDI. The integrated datasets included 1) school attendance (education data 2015–2018), 2) student enrolment and variables (sociodemographic data) 2018, and 3) GSS variables 2018. The GSS employs a three−stage stratified design to select one respondent aged 15+ per household; data were collected via computer−assisted personal interviews (GSS data dictionary) (54).

Data preparation included extracting all IDI datasets on student education, enrollment, school presentations, and the 2018 GSS survey (**Table 1**). Data quality, including integration rate evaluations, was assessed. This included using multiple datasets for the same set of variables to achieve the best matching rates (i.e., minimizing missing global IDs for the linking). In the results, variable suppression (# suppressed due to small counts) was consistent with IDI output rules, according to the Five Safes (4), and was in compliance with output checking.

**Table 1:** Variables included in the study.

School attendance variables	School absenteeism was calculated based on available Term 2 data over the years. Chronic absence rates are defined as missing >30% of school days (measured in half days according to the NZ Ministry of Education). Moderate absence is defined as students missing 20%–30% of school days. Normal presence is defined as students with >80% presence.
Student enrollment variables (sociodemographic data)	Ethnicity, gender (sex), and citizenship
GSS variables (included in the current study)	These provide a basis for cross-domain analysis of social well-being. The included domains in the current study are: • Overall well-being: life satisfaction, sense of purpose, and perceived subjective well-being. • Health: self-rated health status, access to healthcare, and health conditions. • Safety and security: perceptions of safety at home and in the community, and experiences of crime. • Social connections: support networks, loneliness, and frequency of contact with friends and family. • Acceptance of cultural identity: ethnicity, language, and cultural participation. • Trust and institutions: trust in government, police, and other institutions. • Discrimination and inclusion: experiences of discrimination (due to age/skin color/appearance/ethnicity/language/gender/sex/religions/language/gender/religion/disability and social inclusion.

#### 3.2.1 Replicated weights provided in the GSS

The personal weight provided in the GSS is corrected for unequal probabilities of selection, non-response bias, and post-stratification to align with population totals. In addition, the GSS uses replication to generate 100 replicate *bootstrap weights* for robust variance estimation. In the current study, we incorporated the *full sample weight* (gss_pq_person_finalwgt) into the primary estimates and used the *replicated weights* (gss_pq_person_finalwgt_1 to 100) to derive robust standard errors for the regression and path model coefficients.

For BN analyses, the *replicated weights* were also used to generate weighted bootstrap pseudo−samples.

### 3.3 The methodological triangulation framework and hybrid/complementary learning

Guided by the principles of the methodological triangulation framework (55) and hybrid/complementary learning (9, 56), the studies integrated supervised learning and unsupervised learning to strengthen the internal validity and elucidate the complex relations among predictors and outcomes. The use of multiple analytical models with differing assumptions is consistent with methodological triangulation, enhancing internal validity through convergence of evidence.

Our study used supervised learning methods for quantile regression and path analysis, complemented by unsupervised learning and Bayesian network analysis. The supervised learning methods revealed the directional relationships between the response variable (WHO-5) and relevant predictors and confounders. Path analysis further analyzed the interrelationships among the predictors through the specification of exogenous and endogenous variables and their direct and indirect associations with the outcomes. Unsupervised learning and Bayesian network analysis, without imposing *a priori* causal directionality, present data-driven networks among all variables. Through triangulation of the results, convergence (consensus) among the results of these methods provided an internal validity assessment; discrepancies across methods also indicated potential methodological limitations and model-dependent sensitivity (**Figure 1**).

### 3.4 Weighted mixed quantile regressions with repeated measures of the exposure variable—school absenteeism

The response variable WHO-5 mental health well-being was left-skewed; the likelihood-based linear quantile regression models based on the asymmetric Laplace distribution were used. Quantile regression models the median and quantiles without assuming normality. Mixed-effects quantile models included a random intercept for each student to account for repeated absenteeism measures (2015–2018). The fixed effects comprised demographic, family, psychosocial, institutional trust, health, social connection, safety, and discrimination variables.

The R package “lqmm” was used to fit linear quantile mixed models based on the asymmetric Laplace (AL) distribution of random errors. This function computes an estimate of the *τ*th quantile function of the response, conditional on the covariates and random effects. The function maximizes the (log) likelihood of the Laplace regression proposed by Geraci and Bottai (57).

(1) $$\text{Assume }y=\;\mu^{\left(\tau \right)}+\epsilon^{\left(\tau \right)},\;{\text{ϵ}}_{i,j}^{\left(\tau \right)}\sim AL(0,\delta ,\tau ),$$

From Equation 1, AL represents an asymmetric Laplace distribution with location parameter equal to 0, scale parameter δ>0, and skew parameter 0<τ<1 in the fixed model context.

In the random model context,

(2) $$\mu^{\left(\tau \right)}=X\beta^{\left(\tau \right)}+Zu^{\left(\tau \right)}$$

where $\beta^{\left(\tau \right)}$ represents the fixed effect coefficients of *X* and $u^{\left(\tau \right)}$ represents the random effects coefficients of *Z*.

A random intercept model from Equation 2 is when $u^{\left(\tau \right)}\sim N(0,\theta^{\left(\tau \right)}I)$, where *Z* represents the block diagonal matrix, where the diagonal blocks are given by vectors of ones, *I* is an identity

matrix of appropriate dimensions, and $\theta^{\left(\tau \right)}$ is the scale parameter.

Given Equation 1, where ***y*** was the P50—the median of the response variable WHO-5 mental health well-being index in our study—the mixed quantile regressions were fitted and included the repeatedly collected exposure school absenteeism recorded from 2015 to 2018, using the random intercept of the student subject (*Z*), with the fixed effects including the characteristics of students (gender *X*1, NZ citizen *X*2, and ethnicity at level 1 *X*3) and family (family well-being *X*4 and number of dependent children *X*5) from the GSS. Other independent fixed effect variables included from the GSS were institutional trusts (education system) (*X*6), perceived general health conditions (*X*7) and life satisfaction (*X*8), cultural identity acceptance (*X*9), social connections with family (*X*10) and friends (*X*11), and social safety [fear of crime in neighborhood (*X*12) and experience of discrimination (*X*13)]. Model fittings of the full sample weighted model were evaluated using the Akaike information criterion (AIC).

### 3.5 Weighted path model analysis

A path model (58) in the PROC CALIS software in SAS was used to specify the functional relationships between the continuous outcomes (WHO−5 and life worthwhileness) and the endogenous and exogenous continuous variables. The exogenous variables included acceptance of cultural identity, life satisfaction, and perceived general health. A latent institutional trust construct was indicated by trust in education (endogenous within the model), trust in health, trust in parliament, and trust in the police. Robust options were used to address non−normality. Model fit was evaluated using χ (2), the goodness-of-fit index (GFI), the adjusted GFI (AGFI), the root mean square error of approximation (RMSEA), the standardized root mean square residual (SRMR), and the AIC.

In the path model, the functional relationships of the included variables need to be defined. These functional relationships are represented as single-headed paths in the path diagram. The path model defines three sets of variables: outcomes, exogenous variables, and endogenous variables. Outcome variables are defined in the directional path, similar to the response variable in regression, and are presented with arrows pointing to the predictors. Exogenous variables are those variables only included as predictors and never pointed at by a single-headed arrow. Endogenous variables are defined as variables being pointed to by at least one single-headed arrow or serving as an outcome variable in at least one path.

In the current study, these three sets of variables were as follows:

Outcomes:

Subjective mental health well-being: modeled by two observed measures—the WHO-5 mental well-being index and a “perceived life worthwhileness” rating—which reflect the mental well-being and mental health state, respectively.

Exogenous variable:

Social connections: acceptance of cultural identity, a self-reported measure of how easily an individual feels that they can be themselves.

Life satisfaction: a general feeling about life as a whole.

Perceived general health condition—a self-rated general health status (e.g., a single-item rating of overall health).

Endogenous variable in the path:

Institutional trust included four observed measures: trust in the education system (an endogenous variable), trust in the health system, trust in parliament, and trust in the police.

Path analysis investigated the functional relationships among two outcome variables from the GSS—WHO-5 mental well-being (*W*) and perceived life worthwhile (*L*)—and the same set of continuous covariates, which included perceived general health condition ( X7), life satisfaction ( X8), cultural identity ( X9), and trust in the education system ( X6).

Trust in the education system ( X6) was significantly associated with trust in other systems (G1, G2, and G3), with the most variations explained by outcomes *W* and *L*. Therefore, in addition, a latent structure in the path analysis also includes links that point to trust in the education system ( X6) from trusts in other systems (health G1, police G2, and parliament G3).

The pre-specified variance and covariance terms to be estimated in the path model were as follows:

(3) $$(\begin{array}{ccccc} \phi_{W} & \theta_{W,X7} & \theta_{W,X8} & \theta_{W,X9} & \theta_{W,X6}\\ \phi_{L} & \theta_{L,X7} & \theta_{L,X8} & \theta_{L,X9} & \theta_{L,X6}\\ \phi_{X7}\\ \phi_{X8}\\ \phi_{X9}\\ \phi_{X6} \end{array}),$$

(4) $$(\begin{array}{c} \begin{array}{cccc} \phi_{X6} & \theta_{X6,G1} & \theta_{X6,G2} & \theta_{X6,G3}\\ \phi_{G1} & \theta_{G1,G3}\\ \phi_{G2} & \theta_{G2,G3}\\ \phi_{G3} \end{array} \end{array}).$$

Equation 3 is the variance–covariance matrix between outcome variables and exogenous variables and one endogenous variable, trust in the education system. Equation 4 is the variance–covariance matrix between the endogenous variable (trust in the education system) and the other exogenous variables (trust in health, police, and parliament).

#### 3.5.1 Structural paths

The path diagram (**Figure 1**) specified and tested the hypothesis that the independent variables, including social connectedness, perceived health condition, and life satisfaction, were all treated as exogenous predictors; trust in education had a direct influence on the two observed outcome variables (mental well-being and life worthwhileness). Institutional trust (including health, police, and parliament) also had a directional influence on participants’ trust in the education system.

The model-fitting criteria included the absolute indices, which fit measures without referring to any baseline model and do not adjust for model parsimony, including the chi-square test for the model fit with p-value, GFI, and SRMR. Model fitting also included the parsimony index, which adjusts for model complexity, i.e., the number of parameters included [including AGFI, RMSEA, the AIC, and the probability of close fit]. For the RMSEA estimate, convention values under 0.05 indicate good model fit.

### 3.6 Weighted BN structure learnings

BN structure learning is applied to model interconnections among the outcome variables and other continuous/ordinal measures. They were used to construct BN structures that summarize the directional hierarchical structures within the outcome of WHO-5, mental well-being measures *W*, perceived general health X7, and the same set of variables for family (family well-being  X4 and number of dependent children X5), institutional trusts (trust in education X6), life satisfaction  X8, acceptance of cultural identity X9, social connections with family X10 and friends X11, and social safety (fear of crime in neighborhood X12 and experience of discrimination X13).

BN structure learning of the abovementioned scale variables was conducted using the R package “bnlearn” (59). “bnlearn” provides three types of algorithms for structure learning: constraint-based learning, score-based learning, and hybrid learning. These algorithms use different optimization functions to learn the directed graphical structure from the data. For example, hill-climbing of a score-based learning algorithm conducts optimized implementation using score caching, score decomposability, and score equivalence to reduce the number of duplicate tests. TABU search is a modified hill-climbing method to escape local optima by selecting a network that minimally decreases the score function (60). Score-based algorithms (hill-climbing, TABU, and RSMAX2) use network scores to determine edge inclusion, and the constraint-based algorithm uses a conditional dependence test (edges with p-values <0.05) as a significant threshold for edge inclusion.

The five structure learning algorithms included in the current study are as follows:

- Hill-climbing and *TABU search* (score-based)
- Hybrid HPCand *restricted maximization* (hybrid structure)
- Max–min hill-climbing (constraint-based)

The full sample weight (gss_pq_person_finalwgt) was used to create a bootstrap pseudo-sample providing the full sample-weighted BN.

### 3.7 Variance estimation and replicated weights

One hundred replicate−weighted models for regression and path analysis were run. Weighted regression coefficients were summarized across replicates using medians and quantiles. Variances were computed using two replicate−weight variance formulas, and the full sample weight (gss_pq_person_finalwgt) was used for point estimates. For regression and path analysis, the variance based on replicated weights was manually calculated and centered at the full-sample weighted estimate using the Rao–Wu rescaled bootstrap method (61, 62):

$\hat{v}(\hat{\theta })=\frac{1}{T}\overset{100}{\underset{r=1}{\sum }}{\left({\hat{\theta }}_{r}-{\bar{\theta }}^{\left(.\right)}\right)}^{2}$, where ${\hat{\theta }}_{r}$ is the weighted estimator using a replicated weight, ${\bar{\theta }}^{\left(.\right)}$ is the mean of the replicate estimate, and *T* is the number of replicated weights. The replicated weight-based variance accounts for the violation of the independent and identical conditions caused by the complex design.

For BN analyses, replicated weights were used to construct pseudo−populations for 100 bootstrap resamples. Edge stability was summarized by the proportion of replications in which an edge appeared (with >33% considered moderate evidence).In BN analysis, the bootstrap samples were drawn based on the probability derived from replicated weights (47).

All replicated weight-based analyses were performed using the SAS and R programming; R packages “BNlearner” and “lqmm” were included in the replicated weight-based variance estimation.

## 4 Results

### 4.1 Weighted mixed quantile regressions vs. unweighted mixed quantile regression

The weighted quantile regression (based on the full sample weight) identified several factors significantly associated with the WHO−5 mental health well−being index, including ethnicity, trust in the education system, perceived general health, life satisfaction, number of dependent children, acceptance of cultural identity, adequacy of contact with family and friends, and experiences of discrimination in the past 12 months (**Table 2**). School absenteeism was not significantly associated with the WHO−5. The AIC from the mixed quantile regression model, including school absenteeism, was 8,303 (df = 4); including other covariates improved model fitness with a reduced AIC of 7,244 (df = 27). Full-sample-weight mixed quantile regression, including other covariates, had an AIC of 4,150,976 (df = 27).

**Table 2:** Quantile regression for WHO-5 mental well-being index.

Variable description	Unweighted estimates	Std. error	p-values	Full sample weighted estimates	Std. error	p-values	95% CI (lower bound) (replicated weight-1)^&^	95% CI (upper bound) (replicated weight-1)	95% CI (lower bound) (replicated weight-2)^&^	95% CI (upper bound) (replicated weight-2)
Intercept	31.12	11.52	0.009**	25.06	10.69	0.023*	22.90	27.21	22.91	27.21
NZ definition of absenteeism (n = 84) vs. Normal presentation (n = 240)	−0.14	0.56	0.80	0.03	0.40	0.931	−0.16	0.21	−0.15	0.22
Gender: Male (n = 165) vs. Female (n = 165)	0.04	1.41	0.979	0.16	1.41	0.909	−0.31	0.49	−0.26	0.58
NZ citizen (n = 303) vs. Non-citizen (n = 27)	1.53	3.31	0.647	1.65	3.31	0.620	0.81	2.37	0.86	2.44
Ethnicities: Māori (n = 66) vs. European (n = 192)	−0.53	2.20	0.809	−1.38	2.06	0.506	−1.94	−0.90	−1.91	−0.86
Pacific (n = 24) vs. European	−2.04	3.05	0.506	−2.26	2.76	0.417	−2.78	−1.65	−2.83	−1.69
Asian (n = #) vs. European	−0.08	2.86	0.977	−2.45	2.75	0.378	−2.93	−1.86	−3.00	−1.91
Middle Eastern/Latin American/African (MILA) and others (n = #) vs. European	Suppressed#			—	—	—			—	—
Trust in education (0–10)	0.33	0.59	0.576	0.44	0.44	0.320	0.18	1.39	−0.46	1.35
Family well-being (0–10)	0.45	0.60	0.455	0.22	0.51	0.670	−0.45	0.41	−0.41	0.85
Fear of crime (0–10)	−0.48	0.39	0.219	−0.09	0.38	0.812	−0.42	0.46	−0.58	0.40
Perceived health condition##: Very good vs. Excellent	−5.91	1.93	0.004**	−5.47	1.79	0.004**	−6.03	−5.09	−5.97	−4.98
Good vs. Excellent	−9.07	2.20	0.0001***	−10.12	1.96	<0.0001***	−10.74	−9.51	−10.73	−9.51
Fair vs. Excellent	−17.75	2.61	<0.0001***	−18.26	3.01	<0.0001***	−18.90	−17.63	−18.89	−17.62
Poor vs. Excellent	−25.47	4.84	<0.0001***	−26.47	4.79	<0.0001***	−27.46	−25.46	−27.47	−25.48
Life satisfaction (0–10)	3.79	0.55	<0.0001***	4.11	0.61	<0.0001***	3.13	4.65	3.24	4.98
Family with number of dependent children: 1 vs. 0	−0.85	1.83	0.643	**1.53**	1.56	0.332	1.01	1.93	1.06	2.00
Dependent children: 2 vs. 0	0.04	1.89	0.983	0.40	2.42	0.871	−0.08	0.94	−0.12	0.91
Dependent children: 3+ vs. 0	−2.35	2.64	0.377	−0.12	2.51	0.963	−0.77	0.60	−0.80	0.56
Cultural identity##: Easy vs. Hard	8.95	2.73	0.002**	9.49	3.44	0.008**	8.77	10.19	8.79	10.20
Very Easy vs. Hard	8.75	2.63	0.002**	10.00	3.40	0.005**	9.26	10.70	9.28	10.72
Enough contact with family: Too much vs. Just the right amount	2.38	7.91	0.765	**5.32**	7.96	0.507	3.60	7.00	3.62	7.01
Enough contact with family: Not enough vs. Just the right amount	−0.93	8.01	0.908	0.67	7.86	0.933	−1.04	2.34	−1.01	2.35
Enough contact with friends: Not enough vs. Just right	−5.10	1.88	0.009**	−3.42	1.65	0.043*	−3.89	−2.81	−3.98	−2.86
Being discriminated against in the past 12 months: No vs. Yes	−1.67	2.13	0.437	−1.79	2.28	0.436	−2.44	−1.28	−2.39	−1.20

NZ, New Zealand; IDI, Integrated Data Infrastructure.

^#^The result was suppressed due to the small number of participants according to the IDI rule. The total number of students of Asian/Middle Eastern, African, and other ethnicities was 48 (rounded to the nearest base of 3, in accordance with the data protection rule).

^##^Perceived general health and ease of cultural acceptance were reversed so that higher values represent better outcomes (i.e., easiest and excellent received the maximum number).

^&^Replicated weight 1 CI is based on the Rao–Wu method; Replicated weight 2 CI is based on the general STATA method centered at the full sample weighted estimate. *p value < 0.05; ** p value < 0.01; *** p value < 0.005.

For variance estimation in the weighted regression coefficients, after incorporating replicated weights, several covariate associations changed meaningfully based on the 95% confidence intervals. Ethnic group comparisons that were non−significant in the unweighted and full sample weighted analysis became significant in the replicated weight-calibrated results. The weighted differences and their calibrated 95% confidence intervals in the WHO−5 for Māori, Pacific, and Asian groups relative to European students were −1.38 (95% CI (61): −1.94, −0.90), −2.26 (−2.78, −1.65), and −2.45(−2.93, −1.86), respectively. Two types of weighted estimated confidence intervals were all below zero (**Table 2**, 95% CI replicated weight-1,2). A similar shift occurred for trust in education. Students’ trust in the education system was positively associated with the WHO-5; every point increase was associated with a 0.44-point increase in the WHO-5 (95% replicated weight-calibrated CI: 0.18, 1.39).

### 4.2 Weighted path analysis (covariance structure model) vs. unweighted path analysis

#### 4.2.1 Weighted structural relationships

**Figure 2:**
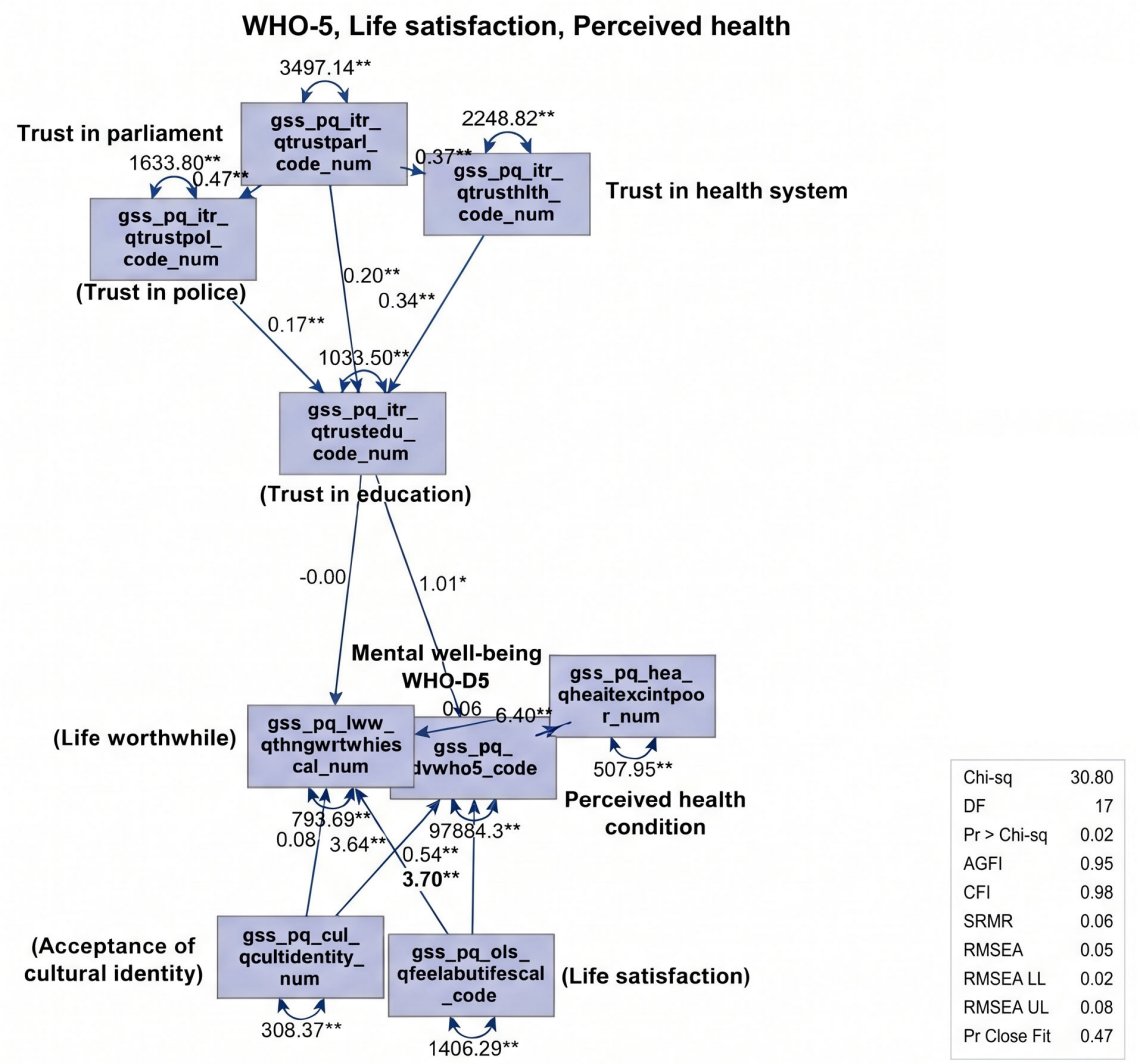
Path diagram: weighted path diagram (based on the full sample personal weight in GSS). *Regressions of variables are represented by one-headed arrows. Regression coefficients are indicated along these one-headed arrows. Variances and covariances among the variables are represented by two-headed arrows. Error variances and covariances are also represented by two-headed arrows.

The full sample weighted path analysis (**Table 3**; **Figure 2**) supported the hypothesized relationships among the key continuous variables—perceived general health, social well−being, trust in the education system, life satisfaction, and acceptance of cultural identity —and the outcome measures (WHO−5 mental health well−being and perceived life worthwhileness). Latent trust constructs incorporated trust in the police, trust in parliament, trust in the health system, and trust in the education system.

**Table 3:** Path model.

Path			Unweighted path coefficient estimates	p-values	Full sample weighted estimates	Standard error	Weighted standardized regression	T value	p-values	95% CI based on the replicated weight variance estimation-1^&^		95% CI based on the replicated weight variance estimation-2^&^	
										Lower bound of confidence interval	Upper bound of confidence interval	Lower bound of confidence interval	Upper bound of confidence interval
WHO-D5 mental well-being index	←	Trust in the education system	0.63 (0.39)	0.106	1.01	0.40	0.109	2.54	0.011*	0.23	1.79	0.57	1.45
Life worthwhile##	←	Trust in the education system	−0.20 (0.03)	0.546	−0.002	0.04	−0.002	−0.05	0.9599	−0.07	0.07	−0.02	0.02
WHO-D5 mental well-being score	←	Life satisfaction	3.76 (0.49)	<0.0001	3.70	0.50	0.342	7.43	<0.0001***	2.72	4.67	3.44	3.95
Life worthwhile	←	Life satisfaction	0.54 (0.04)	<0.0001	0.54	0.04	0.575	12.05	<0.0001***	0.45	0.63	0.51	0.57
WHO-D5 mental well-being score	←	Cultural identity (How easy or hard is it for you to be yourself?)##	2.88 (1.08)	0.008	3.64	1.04	0.158	3.51	0.0004***	1.61	5.67	3.12	4.16
Life worthwhile	←	Cultural identity (How easy or hard is it for you to be yourself?)	0.13 (0.09)	0.148	0.08	0.09	0.041	0.88	0.3815	−0.10	0.26	0.04	0.13
WHO-D5 mental well-being score	←	Perceived general health conditions##	6.02 (0.82)	<0.0001	6.40	0.80	0.356	8.01	<0.0001***	4.84	7.97	6.05	6.76
Life worthwhile	←	Perceived general health conditions	0.03 (0.07)	0.669	0.06	0.07	0.041	0.88	0.3792	−0.08	0.20	0.02	0.10
Trust in education system	←	Trust in the police	0.27 (0.04)	<0.0001	0.17	0.04	0.196	3.97	<0.0001***	0.09	0.26	0.10	0.25
Trust in education system	←	Trust in the parliament	0.22 (0.04)	<0.0001	0.20	0.04	0.265	5.03	<0.0001***	0.12	0.27	0.18	0.21
Trust in education system	←	Trust in the health system	0.28 (0.04)	<0.0001	0.34	0.04	0.406	9.09	<0.0001***	0.27	0.41	0.31	0.37
Trust in health system	←	Trust in the parliament	0.45 (0.04)	<0.0001	0.37	0.04	0.421	8.39	<0.0001***	0.29	0.46	0.34	0.40
Trust in police	←	Trust in the parliament	0.39 (0.04)	<0.0001	0.47	0.04	0.568	12.49	<0.0001***	0.40	0.55	0.45	0.50

^##^Perceived general health, life worthwhileness, and ease of cultural acceptance were reversed in the order of their coding so that higher values represent better outcomes (i.e., easiest and excellent have the maximum number).

^&^Replicated weight 1 CI is based on the Rao–Wu method; Replicated weight 2 CI is based on the general STATA centered at the full sample weighted estimate. *p value < 0.05; ** p value < 0.01; *** p value < 0.005.

Perceived general health, life satisfaction, trust in the education system, and acceptance of cultural identity all showed significant positive effects on mental health well-being (WHO−5). Life satisfaction was the only predictor positively associated with perceived life worthwhileness. Within the latent trust structure, trust in the police, trust in parliament, and trust in the health system all contributed positively to trust in the education system.

While the unweighted model identified similar directional relationships (with the exception of trust in education), several coefficients—particularly for perceived health, life satisfaction, and acceptance of cultural identity—were attenuated compared to the full sample weighted results. Both models demonstrated a strong overall fit (weighted model: CFI = 0.98, AGFI = 0.95, RMSEA = 0.05, SRMR = 0.06).

For replicated weight calibration variance estimation, replicate−weighted models (100 runs) yielded median p−values of 0.020 (0.017–0.023), GFI of 0.979 (0.979–0.980), and RMSEA of 0.05 (0.049–0.051), which supported model robustness.

### 4.3 Replicated BN learning structure vs. unweighted BN learning structure

**Figure 3:**
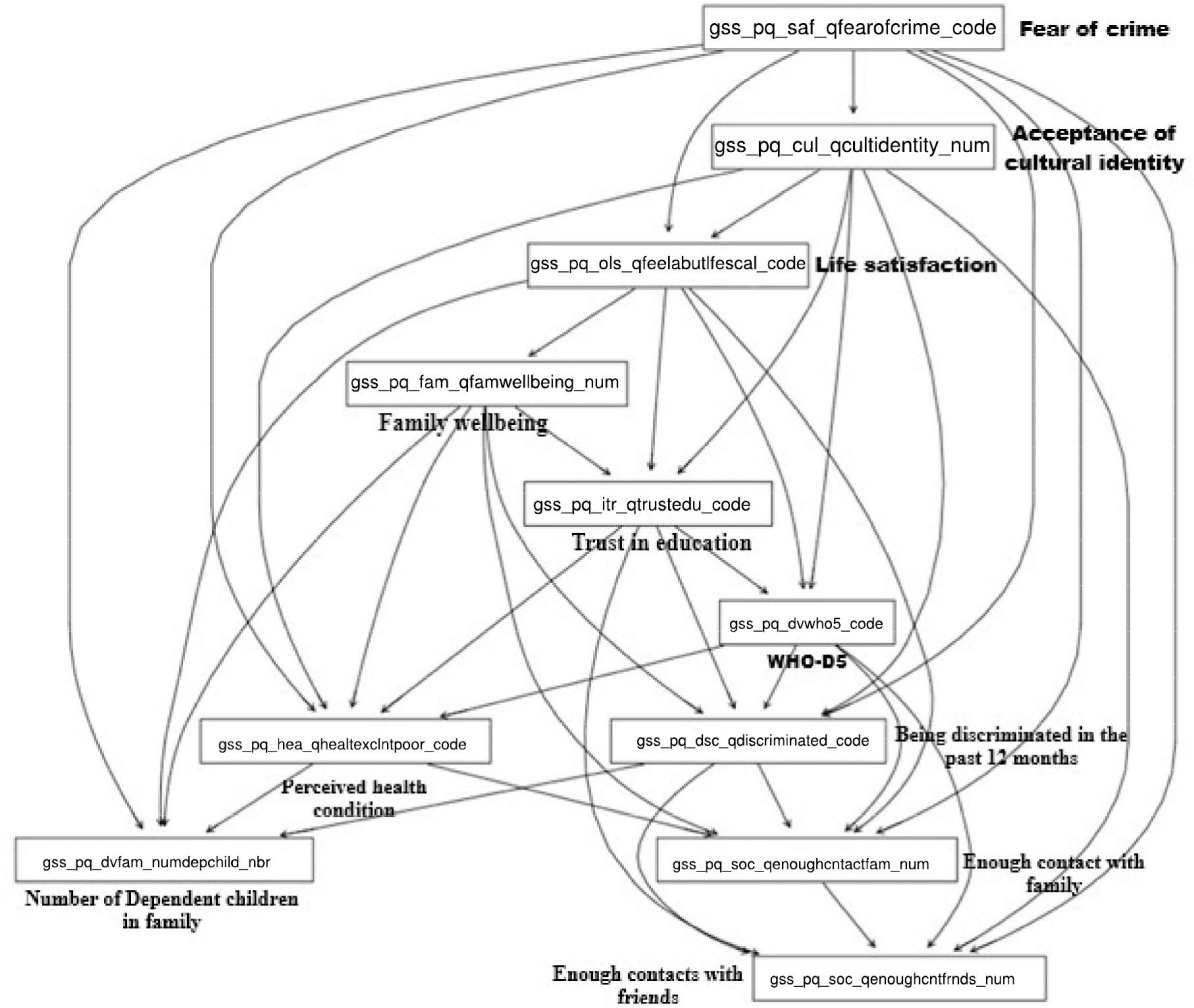
Full weighted TABU BN.

**Figure 4:**
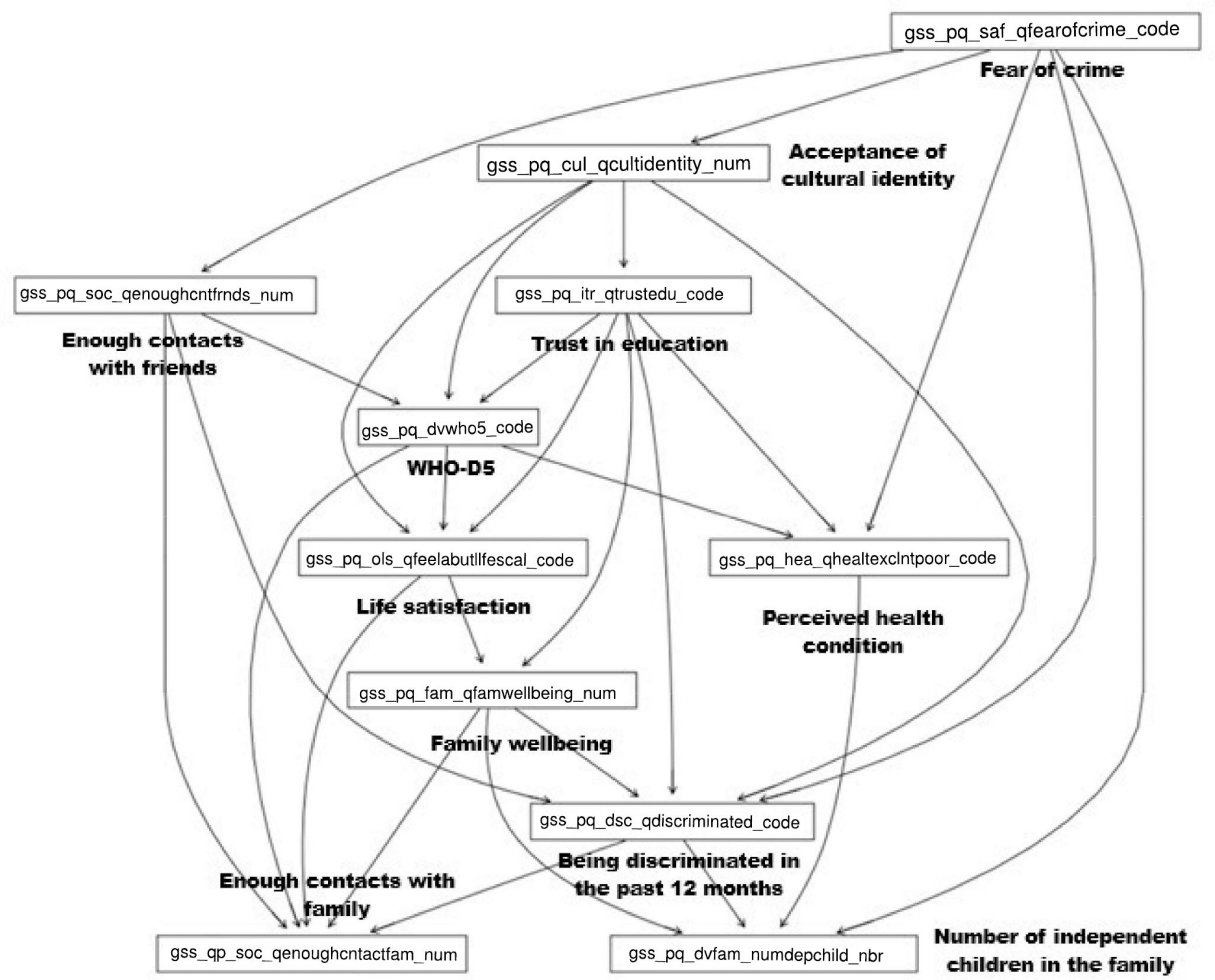
Full weighted MMHC BN.

The full sample weighted BN analysis (**Figure 3**) presents the same significant connections (edges) in the TABU and the max–min hill-climbing (MMHC) algorithm (**Figure 4**) between trust in education, cultural identity acceptance, and WHO-5, with BN coefficients (0.1347 and 0.2032, and 0.2590 and 0.4300, respectively). Life satisfaction was significant in TABU but not in MMHC, and connection with friends was significant in MMHC but not in TABU.

In the replicated, weight-calibrated BN analysis (**Table 4**), cultural identity acceptance and trust in the education system were the most frequent significant predictors of the WHO-5 score across all BN algorithms (HC, TABU, and MMHC). Ease in accepting cultural identity and trust in education systems were identified as significantly connected in more than 25% of the 100 bootstrap replicate results. The TABU algorithm also identified that overall life satisfaction (on a 0–10 scale) was a positive contributor to mental health well-being.

**Table 4:** Summary of the BN analysis results from 100 bootstrap resamples via replicate weights.

a. For WHO-D5 mental well being							
Variable	Algorithm	Number of times identified as a factor influencing WHO-D5 mental well-being out of 100	Mean BN coefficient	Std. dev.	Median BN coefficient	Lower quartile	Upper quartile
Number of dependent children in the household	HC	1	0.065	—	0.065	0.065	0.065
	TABU	17	0.058	0.017	0.065	0.05	0.069
How easy is it to accept your culture as part of who you are?	HC	26	0.403	0.044	0.415	0.402	0.42
	TABU	36	0.342	0.087	0.399	0.244	0.416
	MMHC	28	0.419	0.011	0.42	0.409	0.425
Have you been discriminated against in the last 12 months?	TABU	1	−0.093	—	−0.093	−0.093	−0.093
Family well-being: How would you rate your family's overall well-being?	TABU	2	0.032	0.011	0.032	0.025	0.04
Perceived general health rating: Excellent to Poor scale#	TABU	15	0.667	0.069	0.706	0.619	0.717
Trust in the education system (0–10 scale)	HC	22	0.191	0.02	0.196	0.19	0.2
	TABU	29	0.163	0.035	0.183	0.131	0.197
	MMHC	28	0.198	0.008	0.197	0.192	0.203
Overall life satisfaction (0–10 scale)	HC	2	0.363	0.021	0.363	0.348	0.379
	TABU	29	0.414	0.041	0.414	0.379	0.46
Fear of crime in your area (0–10 scale)	HC	5	0.037	0.013	0.031	0.027	0.049
	TABU	4	0.012	0.052	0.035	−0.017	0.041
Contact with friends: Is it enough, too much, or not enough?	HC	3	−0.343	0.043	−0.348	−0.384	−0.298
	TABU	6	−0.366	0.041	−0.373	−0.384	−0.348
	MMHC	24	−0.36	0.029	−0.354	−0.383	−0.338
Contact with family: Is it enough, too much, or not enough?	HC	1	−0.397	—	−0.397	−0.397	−0.397
	TABU	2	−0.344	0.074	−0.344	−0.397	−0.292
b. For perceived general health conditions							
Variable	Algorithm	Number of times identified as a factor influencing perceived general health conditions out of 100	Mean	Sth. dev.	Median	Lower quartile	Upper quartile
Number of dependent children in the household	HC	7	−0.037	0.009	−0.037	−0.046	−0.03
	TABU	20	−0.039	0.011	−0.038	−0.047	−0.032
	MMHC	20	−0.039	0.011	−0.038	−0.047	−0.032
How easy is it to accept your culture as part of who you are?#	HC	23	0.039	0.008	0.041	0.032	0.045
	TABU	25	0.027	0.04	0.037	0.03	0.044
	MMHC	25	0.027	0.04	0.037	0.03	0.044
WHO-5 mental well-being score	HC	95	−0.303	0.009	−0.303	−0.308	−0.297
	TABU	85	−0.302	0.009	−0.302	−0.307	−0.296
	MMHC	85	−0.302	0.009	−0.302	−0.307	−0.296
Family well-being: How would you rate your family's overall well-being?	HC	25	0.039	0.006	0.039	0.036	0.045
	TABU	31	0.04	0.006	0.04	0.034	0.045
	MMHC	31	0.04	0.006	0.04	0.034	0.045
trust in the education system (0–10 scale)	HC	44	−0.035	0.005	−0.035	−0.039	−0.031
	TABU	51	−0.038	0.012	−0.035	−0.041	−0.032
	MMHC	51	−0.038	0.012	−0.035	−0.041	−0.032
Overall life satisfaction (0–10 scale)	HC	8	−0.027	0.009	−0.023	−0.036	−0.019
	TABU	12	−0.039	0.039	−0.028	−0.037	−0.02
	MMHC	12	−0.039	0.039	−0.028	−0.037	−0.02
Fear of crime in your area (0–10 scale)	HC	35	0.053	0.016	0.053	0.039	0.061
	TABU	34	0.051	0.012	0.053	0.043	0.06
	MMHC	34	0.051	0.012	0.053	0.043	0.06
Contact with family: Is it enough, too much, or not enough?	HC	1	0.064	—	0.064	0.064	0.064
	TABU	1	0.041	—	0.041	0.041	0.041
	MMHC	1	0.041	—	0.041	0.041	0.041

^##^Perceived general health and ease of cultural acceptance were reversed so that higher values represent better outcomes (i.e., easiest and excellent have the maximum number). Contact with friends and family with a higher value represents a worse connection.

a. For WHO-D5 mental well-being.

b. For perceived general health conditions.

BN, Bayesian network.

For perceived general health (higher values indicate poorer health), the most frequently detected predictors included mental health well-being (WHO−5), trust in education, fear of crime, family well−being, and acceptance of cultural identity. Directional interpretation concluded that poorer mental health, lower trust, greater fear of crime, and lower family well−being were consistently associated with worse perceived health across bootstrap replications.

### 4.4 Simulation of data experiments and their results of weighted BN structure learning vs BN structure learning

Simulations (**Table 5**) were conducted with different experimental parameters of *p* (50 or 100) and sample size *n* (50, 500, and 1,000) to compare different BN algorithms in a sparse network (63) (with an edge-to-density ratio <0.1). Both unweighted and weighted methods (**Table 5**) were used. For each experiment, 100 replications were used. The simulation results showed that the TABU algorithm, using weighted and unweighted methods, always had a better detection rate for true relations than MMHC and RSMAX2, but the latter hybrid algorithms had achieved better precision, with a structure-based measure balancing both true-positive and false-positive rates. HC had a similar performance to TABU in the majority of simulations. MMHC and RSMAX2 had a similar performance in the majority of simulations. They both had much lower true and false detection rates.

**Table 5:** Simulation results for BNs.

Data presented is % of true positive (TP), false positive (FP) and precision (P)	n=300		unweighted				weighted			
			HC	TABU	MMHC	RSMAX	HC	TABU	MMHC	RSMAX
Data 1	p = 50	TP*	19.8%	29.5%	0.0%	0.0%	15.7%	16.5%	1.1%	1.2%
		FP*	5.4%	8.3%	0.0%	0.0%	8.4%	8.8%	0.3%	0.3%
		P** = TP/(TP + FP)	78.57%	78.04%	NA	NA	65.15%	65.22%	78.57%	80.00%
Data 2	p = 100	TP	56.8%	57.4%	0.2%	0.2%	39.6%	39.7%	0.7%	0.8%
		FP	19.9%	20.0%	0.1%	0.1%	20.2%	20.2%	0.3%	0.4%
		P**	74.05%	74.16%	66.67%	66.67%	66.22%	66.28%	70.00%	66.67%
Data 3	p = 200	TP	14.9%	15.0%	0.0%	0.0%	–	–	–	–
		FP	2.8%	2.8%	0.0%	0.0%	–	–	–	–
		P**	84.18%	84.27%	NA	NA				
	n=500		unweighted				weighted			
			HC	TABU	MMHC	RSMAX	HC	TABU	MMHC	RSMAX
Data 4	p = 50	TP	25.3%	34.5%	0.1%	0.1%	12.9%	14.4%	0.9%	0.9%
		FP	9.7%	13.0%	0.0%	0.0%	6.3%	7.0%	0.3%	0.2%
		P**	72.29%	72.63%	100.00%	100.00%	67.19%	67.29%	75.00%	81.82%
Data 5	p = 100	TP	69.1%	69.9%	0.1%	0.1%	57.5%	57.6%	0.8%	1.0%
		FP	37.1%	37.6%	0.2%	0.2%	32.5%	32.5%	0.3%	0.5%
		P**	65.07%	65.02%	33.33%	33.33%	63.89%	63.93%	72.73%	66.67%
Data 6	p = 200	TP	41.7%	41.9%	0.0%	0.1%	–	–	–	–
		FP	7.0%	7.1%	0.0%	0.0%	–	–	–	–
		P**	85.63%	85.51%	NA	NA				
	n=1000		unweighted				weighted			
			HC	TABU	MMHC	RSMAX	HC	TABU	MMHC	RSMAX
Data 7	p = 50	TP	39.6%	49.3%	0.4%	0.4%	20.7%	23.1%	0.8%	0.9%
		FP	24.0%	27.5%	0.1%	0.1%	11.1%	12.4%	0.2%	0.2%
		P**	62.26%	64.19%	80.00%	80.00%	65.09%	65.07%	80.00%	81.82%
Data 8	p = 100	TP	70.2%	71.5%	0.2%	0.3%	64.9%	65.1%	0.5%	0.6%
		FP	61.8%	62.5%	0.1%	0.3%	44.6%	44.7%	0.3%	0.4%
		P**	53.18%	53.36%	66.67%	50.00%	59.27%	59.29%	62.50%	60.00%
	p = 200	TP	84.2%	84.2%	0.1%	0.1%	–	–	–	–
		FP	42.3%	42.3%	0.0%	0.0%	–	–	–	–
		P**	66.56%	66.56%	100.00%	100.00%				

*TP = the number of detected true/total number of actual true directional connections to the simulated outcome Y. The other variables included similar structures to the integrated data with independent explanatory variables (X1–X50/100/200) and correlated variables Z1–Z5/10 and Y, Y2, Y3, and Y4. .

*FP = the number of detected false/total number of actual false directional connections to the simulated outcome Y.

**P/precision = TP/(TP + FP) represents the rate of correct edges discovered across all edges discovered.

The simulation results were used to assist interpretations from the different BN algorithms in the previous section on replicated weighted BN results in the IDI dataset.

## 5 Discussion

This study adopted a methodological triangulation framework, using complementary learning to combine supervised methods (quantile regression and covariance-based path analysis) with unsupervised data-driven BN analysis to examine factors associated with students’ mental well−being, perceived life worthwhileness, and perceived general health within the integrated GSS–education dataset. Across models, perceived general health, acceptance of cultural identity, institutional trust (education), and life satisfaction were consistently associated with mental health well-being (WHO-5). BN analyses of perceived health highlighted additional roles for fear of crime and family well−being.

Quantile regression was used to identify variables associated with the WHO-5 outcome, with an aim to test the association between school absenteeism and the outcome when other factors were also accounted for. Path analysis provided a structural model that tested the directional associations between the WHO-5, perceived life worthwhileness, psychosocial factors, and family factors simultaneously. A latent cluster of correlated trust variables (as part of psychosocial factors) was modeled in the covariate structure, in which participants’ trust in the education system was an endogenous variable explaining the largest amount of variation in the outcomes. Bayesian network analysis provided the entire directional network based on the data; the results of the outcomes (WHO-5 and perceived general health) were compared with results obtained using supervised learning models. The three types of models reached a consensus that perceived health condition, ease of accepting cultural identity, trust in the education system, and overall life satisfaction are all positively contributing to students’ mental health well-being (WHO-5). Perceived life worthwhileness was correlated with the outcomes (WHO-5) but was only connected to overall life satisfaction. BN models for perceived general health revealed similar contributing factors to the WHO-5; in addition, fear of crime in the area and the family’s overall well-being also contributed to perceived general health. The consensus on other positive factors attributed to the WHO-5 from replicated weight-calibrated results of both regression and BN analysis was social connections with friends.

Discrepancies in the findings between the replicated weight-calibrated median quantile regression and the BN analysis were shown in discrimination experienced in the past 12 months and connection with family, which were significant in the regression analysis but were not identified in the BN analysis.

In the full sample weighted regression methods, all significant results had consistency in their p values and 95% confidence intervals, compared to those derived using replicated weights. However, there were inferential differences in some p-value non-significant results. For example, the replicated weight-calibrated 95% confidence interval suggested significant differences between Māori, Pacific, Asian, and European ethnic groups; trust in education was significantly associated with the WHO-5 in one of the replicated weight results (Rao–Wu method). When contrasting these discrepancies with BN and path analysis, we concluded that perceived health condition, ease of accepting cultural identity, and trust in the education system were not method-dependent, while discrimination experienced in the past 12 months and connection with family were method-sensitive and warrant future research.

Although both BNs and replicated weighting are improvement techniques that increase the robustness of complex survey analysis, there are methodological developments that are yet to be considered. In fact, pathway analysis—often conducted using techniques such as mediation analysis or structural equation modeling—enables researchers to communicate directly or indirectly related variable relationships and to test hypotheses about directional associations (64).

As is evident in the available literature, incorporating weights for replicate analysis in pathway analysis leads to correct coverage assessment and proper confidence intervals (65). Although involving replicates adds model analysis considerations, methodological integrity guarantees a population-level portrayal in pathway analysis. The need to incorporate weights into both pathway analysis and population-level frameworks, such as BNs (47), adds to improved credibility in population-level portrayal in larger data integration systems, such as the IDI in New Zealand. The current study included replicated weights into three different models to account for the complex survey design. The weighted results provided different point estimates, and significant inference was indicated by the weighted confidence intervals of several predictors in these models. Replicated weight estimates change ethnic comparisons and discrimination associations relative to unweighted analyses, which emphasize the design effect due to unequal selection probabilities or clustering in the sampling.

The BN results using actual data revealed that TABU has a better structure recovery rate than HC, which is consistent with that of Song et al. (66), who reported that TABU BN recovers a richer dependency structure and performs better than HC. In the comparison between TABU and the hybrid algorithm (e.g., MMHC), a recent review of BN structure learning algorithms (25) reports that MMHC and RSMAX (the two hybrid algorithms) outperformed the pure score-based search, such as TABU/HC (score-based alone), in both accuracy and robustness, as measured by graph structure scoring (e.g., precision and recall) and inference-based evaluation (e.g., Bayesian Information Criterion (BICC)), particularly in medium-to-large networks (p = 200 to 1,000s). In the current study (with a small network), both TABU and the hybrid algorithm (MMHC) provided a similar structure in the weighted results for perceived health, but more discrepancy in the weighted results for the WHO-5. The simulations covered the same range of network sizes, only conducted in small-to-medium networks (p = 20–200), providing consistent results that suggested that weighted MMHC and RSMAX2 were better in terms of precision, balancing both true positives and false positives more effectively than TABU, which had higher true-positive rates than the hybrid algorithms (MMHC and RSMAX2). Weighted results were better using BN’s algorithms MMHC and RSMAX2, although both algorithms had lower true-positive detection rates than TABU and HC. A potential reason weighted results were not always better is that the simulated weight variable did not include non-response in sampling weight construction. This simulation mimicked sampling that occurred only among 70% of the respondents, and the weight variable was a base weight only reflecting the inclusion probability of a cluster. These could be addressed using post-stratification weights in real data. These simulations are provided for comparison between different algorithms, not for comparing unweighted vs. weighted results, which has already been well established in survey methodology (67–69).

Compared to stage I of the wider study, the cohort included in the present study was in the normal school presentation group. This cohort did not have any hospital admissions in the following years (2019–2021); in stage I, there were approximately 3,000 cases identified from the same study population from which the cohort was drawn, and school absenteeism was found to be a significant early marker of suicidality (70). This could indicate that the cohort in the current study, those who participated in GSS 2018, is a subset of the wider study population and comprises healthier students. Among these students, the school presentation appeared to be in the normal range and was not associated with their mental health well-being (WHO-5), which was also in the normal range (mean 65 and median 68), and WHO-5 < 50 was considered low well-being (71–73). Although there were other factors associated with these students’ mental health well-being, school presentation was not one of these markers in this cohort.

Our findings align with a growing evidence base that strengthening the cultural identity of rangatahi Māori and their access to cultural resources (including te reo Māori) is associated with better well-being and fewer depressive symptoms, even after accounting for demographics and deprivation; experiences of ethnic discrimination were strongly associated with poorer outcomes (74). Cultural embeddedness (including whole-school normalization and competency in te reo Māori, tikanga, and cross-cultural awareness) strengthens connection, belonging, and engagement at school (75), and it longitudinally predicts coping and well-being among Māori youth (75) (including from the current study). Together, growing evidence (including from the current study) clearly emphasize the need for education and mental health policies that demonstrate belonging and embed identity, language and culture to support learning and mental well-being. Our findings particularly demonstrate that cultural supports for young people, including te reo Māori, are not “nice to have” but rather offer a protective function against psychological distress and may function as a population‑level mental health promotion strategy for young people, particularly Māori. The study limitations are summarized as follows. First, the integrated data did not include any cases of hospitalization due to suicidality. This result is not unexpected because participants who are likely to respond to a survey are likely to be in good health. Students with a history of hospitalization due to suicidality may be on the most severe end of the poor mental health spectrum and are less likely to respond to surveys (76). Second, the replicated weight calibration used to address potential bias could not fully address non-response biases, which would require further investigation, such as adding a post-stratification approach. Third, the replicated weight BN analysis did not provide the empirical distribution of inference-based statistics (AIC and BIC) in the current programs. Finally, the current study was based on integrated school administration data and a survey, which limits the age range to students 15 years and older.

## 6 Conclusion

Using triangulation in different analytical models provides us with a good strategy to achieve internal validity. Complementary supervised and unsupervised learning methods enhance our confidence in the results obtained through convergence and provide us with information about the model-dependent discrepancies.

- The pathway covariance structure model is an advanced parametric method that allows us to test directional relationships between outcomes and predictors, where an endogenous variable is modeled as an outcome variable in at least one path while also being a predictor for the other outcomes.
- The BN method has more freedom and is data-driven. However, different algorithms achieve different results, which can make interpretation difficult. It requires careful testing across different algorithms to reach a consensus.

Integration across quantile regression, pathway, and BN analysis thus offers rigorous methods to detect complex inter-relationships in mental health and survey data, especially when used in population-level datasets, such as those being harnessed in New Zealand’s IDI. Applying replicated weights as a design-based approach to calibrating complex sampling weights is important, especially in studies with small samples and large dimensions.

In future research, a program for complex survey analysis could include weighted BN coefficient aggregation for the final graph and provide guidelines for both graph scoring and inference-based statistics (e.g., AIC and BIC) in weighted BN algorithms. Incorporating replicated weights for aggregation summary and model fitness criteria in the current R qlmm function will also be advantageous for quantile regression. Further methodological triangulation could integrate linear regression (e.g., providing prior coefficients) with BN structure learning (moving from supervised to unsupervised learning) or vice versa, from BN learning discovery to linear regression validation (moving from unsupervised to supervised learning).

## Data Availability

The original contributions presented in the study are included in the article/supplementary material, further inquiries can be directed to the corresponding author/s.
